# Radiation exposure of a mobile 3D C-arm with large flat-panel detector for intraoperative imaging and navigation - an experimental study using an anthropomorphic Alderson phantom

**DOI:** 10.1186/s12880-020-00495-y

**Published:** 2020-08-14

**Authors:** Yashar Naseri, Ulrich Hubbe, Christoph Scholz, Johannes Brönner, Marie T. Krüger, Jan-Helge Klingler

**Affiliations:** 1 Department of Neurosurgery, Medical Center - University of Freiburg, Faculty of Medicine, University of Freiburg, Breisacher Str. 64, 79106 Freiburg, Germany; 2 Department of Neurosurgery, Cantonal Hospital St. Gallen, St. Gallen, Switzerland; 3 Helmholtz Zentrum München, German Research Center for Environmental Health, Individual Monitoring Service, Munich, Germany

**Keywords:** 3-dimensional, C-arm, Dosimetry, Intraoperative imaging, Minimally invasive surgery, Navigation, Phantom, Radiation exposure, Spine

## Abstract

**Background:**

Intraoperative 3-dimensional (3D) navigation is increasingly being used for pedicle screw placement. For this purpose, dedicated mobile 3D C-arms are capable of providing intraoperative fluoroscopy-based 3D image data sets. Modern 3D C-arms have a large field of view, which suggests a higher radiation exposure. In this experimental study we therefore investigate the radiation exposure of a new mobile 3D C-arm with large flat-panel detector to a previously reported device with regular flat-panel detector on an Alderson phantom.

**Methods:**

We measured the radiation exposure of the Vision RFD 3D (large 30 × 30 cm detector) while creating 3D image sets as well as standard fluoroscopic images of the cervical and lumbar spine using an Alderson phantom. The dosemeter readings were then compared with the radiation exposure of the previous model Vision FD Vario 3D (smaller 20 × 20 cm detector), which had been examined identically in advance and published elsewhere.

**Results:**

The larger 3D C-arm induced lower radiation exposures at all dosemeter sites in cervical 3D scans as well as at the sites of eye lenses and thyroid gland in lumbar 3D scans. At ​​male and especially female gonads in lumbar 3D scans, however, the larger 3D C-arm showed higher radiation exposures compared with the smaller 3D C-arm. In lumbar fluoroscopic images, the dosemeters near/in the radiation field measured a higher radiation exposure using the larger 3D C-arm.

**Conclusions:**

The larger 3D C-arm offers the possibility to reduce radiation exposures for specific applications despite its larger flat-panel detector with a larger field of view. However, due to the considerably higher radiation exposure of the larger 3D C-arm during lumbar 3D scans, the smaller 3D C-arm is to be recommended for short-distance instrumentations (mono- and bilevel) from a radiation protection point of view. The larger 3D C-arm with its enlarged 3D image set might be used for long instrumentations of the lumbar spine. From a radiation protection perspective, the use of the respective 3D C-arm should be based on the presented data and the respective application.

## Background

Spinal surgery and instrumentation often comprise placement of implants without direct view of their trajectory or proximity to adjacent neurovascular structures [1]. Therefore intraoperative imaging is indispensable for the accurate placement of implants in spine surgery. The use of fluoroscopy has increased with the rising number of stabilization procedures. Especially minimally invasive spinal surgery tends to increase the need for fluoroscopy. Three-dimensional (3D) imaging devices are capable to acquire intraoperative 3D image sets to facilitate intraoperative 3D navigation and to improve the accuracy of screw placement [2]. For this, mobile 3D C-arms use the principle of digital volume tomography to create a 3D image set out of multiple 2D fluoroscopic images [3]. However, as imaging with C-arms is based on ionizing radiation, the use of fluoroscopy or 3D imaging modalities is associated with the risk of considerable radiation exposure to the patient, surgeon and surgical staff [4]. This is associated with potential morbidity including skin erythema, cataract formation, or development of malignancies [5].

This study was conducted to assess the radiation exposure of the state-of-the-art C-arm Vision RFD 3D with a large flat-panel detector (30 × 30 cm) using an anthropomorphic phantom and to compare it with the results of the C-arm Vision FD Vario 3D with a standard flat-panel detector (20 × 20 cm) that we have reported elsewhere [3].

## Methods

Mobile 3D C-arms perform an automated orbital rotation around the patient and acquire a 3D image set consisting of successive 2D fluoroscopic images. We measured the radiation exposure of the Vision RFD 3D (Ziehm Imaging, Nuremberg, Germany) to create 3D image sets as well as standard fluoroscopic images on the cervical and lumbar spine. This device has a large flat-panel detector of 30 × 30 cm. We then compared the radiation exposure to the Vision FD Vario 3D (Ziehm Imaging) with a standard flat-panel detector of 20 × 20 cm that we have previously investigated using the identical protocol and which results we have published elsewhere [3].

### 3D C-arm devices

#### Vision RFD 3D

**Fig. 1:**
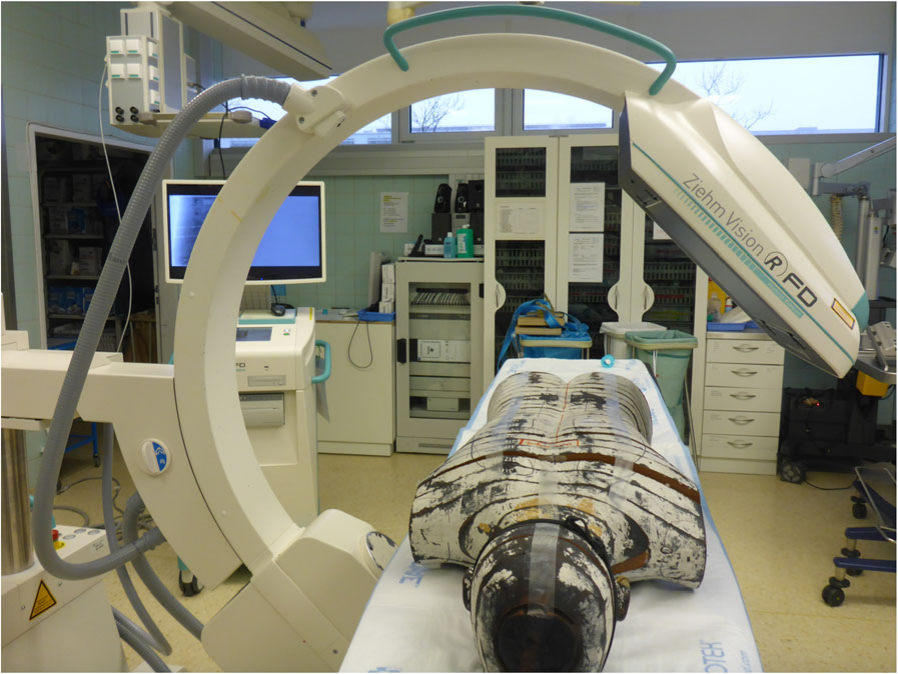
An Alderson phantom was placed in prone position on a radiolucent operating table. The radiation exposure was measured at various sites (eye lenses, thyroid gland, female and male gonads) using dosemeters

The Vision RFD 3D provides a 3D image set using a large 30 × 30 cm digital flat-panel detector and 180-degree scanning arc (Fig. 1) consisting of an automated initial 7.5° linear, a 165° rotating and a final 7.5° linear movement around the patient. The scan takes 48 s to acquire approximately 390 fluoroscopic images. Subsequently, a 3D image set with resolution of 320 voxels and an edge length of 16 cm is displayed on the screen (volume of 3D image set: 4096 cm^3^) [6].

#### Vision FD Vario 3D

The Vision FD Vario 3D has a 20 × 20 cm digital flat-panel detector. During the scanning arc of 135 degrees, 110 fluoroscopic images are captured in 64 s. Subsequently, a 3D image set with resolution of 512 voxels and an edge length of 12.8 cm is provided (volume of 3D image set: 2097 cm^3^) [7].

### Measurement of radiation exposure

The measurement setup is identical to the publication by Klingler et al. [3].

**Fig. 2:**
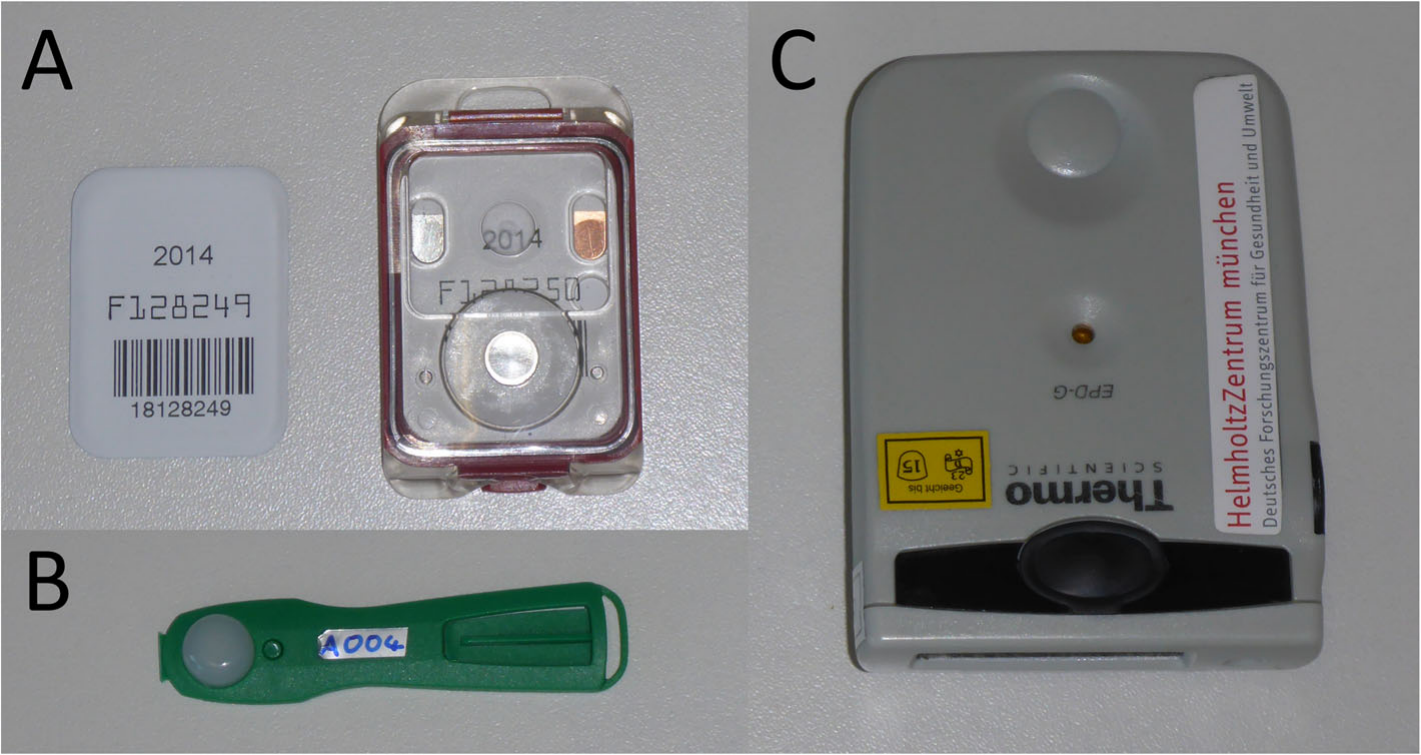
Overview of used dosemeter types. Film dosemeter with cassette (**a**), eye lens thermoluminescence dosemeter (**b**), electronic personal dosemeter with digital display (**c**)

An anthropomorphic Alderson phantom was used to measure the radiation exposure of the C-arm. The humanly shaped phantom consists of a human skeleton embedded in tissue-equivalent material. Positioned in prone position, the phantom was placed on a radiolucent operating table (Fig. 1). The test setup consisted of 1) 10 3D scans or 2) 200 lateral and 200 anterior-posterior fluoroscopic images. In each setup, three film dosemeters each were attached to the surface of the phantom at the thyroid gland, female and male gonads [AWST-FILM-GD 60, *H*_*p*_(10); Helmholtz Zentrum München German Research Center for Environmental Health, Personal Monitoring Service, Munich, Germany, Fig. 2a. Additionally, three eye lens thermoluminescence dosemeters were placed at the position of the eye lens [EYE-D™, *H*_*p*_(3); Radcard, Krakow, Poland, Fig. 2b.

Ten automated 3D scans were performed on the cervical and lumbar spine with the X-ray beam centered on the phantom’s vertebral body of C4 and L3, respectively.

For assessment of the radiation exposure of fluoroscopic images, the flat-panel detector was positioned in 25 cm distance from the surface of the phantom for anterior-posterior projection and in 10 cm distance for lateral projection, respectively. Likewise, the X-ray beam was centered on the vertebral body of C4 and L3, respectively. To simulate the surgeon’s position, further film dosemeters (GD 60) were attached to the generator and flat-panel detector of the C-arms additionally to the above-quoted locations. Furthermore, electronic personal dosemeters [EPD Mk2; Thermo Scientific, Schwerte, Germany, *H*_*p*_(10) mode, Fig. 2c] were placed above and under a lead apron (lead equivalent front part Pb 0.7 mm; Mavig, Munich, Germany) at the surgeon’s position right next to the C-arm generator to assess the radiation exposure. In all measurements the C-arm was set to automatic exposure control with no application of collimation. For background subtraction, five film and five EYE-D™ dosemeters were positioned outside the operating room. The Helmholtz Zentrum München (Munich, Germany) provided and evaluated all dosemeters.

According to Physics-related difficulties in dosemetric assessment of radiation exposure, such as appropriate background subtraction, lower detection limits and therefore measurement accuracy, uncertainty models were used to finally assume a lower detection limit of 30 μSv for eye lens dosemeters and 44 μSv for film dosemeters for each test series [8].

### Statistical analysis

At each dosemeter location, the average radiation exposure for one 3D scan was calculated.

For each test setup of fluoroscopic images with 200 lateral and 200 anterior-posterior fluoroscopic images, the average radiation exposure of the dosemeters at each location was calculated to determine the mean radiation exposure for one representative fluoroscopic image. Differences in mean scores of radiation exposure (Vision RFD 3D versus the results of the Vision FD Vario 3D published elsewhere [3]) were compared with the two-tailed Student’s t test. A *p*-value < 0.05 was considered to be statistically significant.

## Results

### Radiation exposure for 3D image sets

In cervical 3D scans, all dosemeter sites showed numerically lower radiation exposures for the Vision RFD 3D compared with the Vision FD Vario 3D (Table 1). Statistically significant lower radiation exposure was observed for the Vision RFD 3D at the thyroid gland (2173.3 versus 4405.2 μSv, *p*-value < 0.05).

**Table 1:** Mean radiation exposures with standard deviations in μSv for acquisition of one cervical and one lumbar 3D image set, respectively. For each reading of a test series that was below the lower detection limit of the dosemeters, the value of the lower detection limit (44 μSv for film dosemeters) was used leading to the final estimation of a maximum radiation exposure of 4.4 μSv (44 μSv/10)

	Eye lenses	Thyroid gland	Female gonad	Male gonad
**Cervical 3D image set**				
Vision RFD 3D	287.2 ± 28.8	2173.3 ± 302.9^a^	< 4.4	< 4.4
Vision FD Vario 3D ^b^	294.1 ± 19.5	4405.2 ± 133.8	< 4.4	< 4.4
**Lumbar 3D image set**				
Vision RFD 3D	11.0 ± 1.1^a^	9.8 ± 2.0^a^	6196.5 ± 490.6^a^	66.9 ± 16.4
Vision FD Vario 3D ^b^	22.5 ± 2.4	32.8 ± 6.8	1368.6 ± 501.9	32.3 ± 23.4

^a^indicate *p*-values < 0.05 in comparison to the Vision FD Vario 3D

^b^Results of Klingler et al. [3]

In lumbar 3D scans, statistically significant lower radiation exposure was observed for the Vision RFD 3D at the thyroid gland (9.8 versus 32.8 μSv, *p*-value < 0.05) as well as at the eye lenses (11.0 versus 22.5 μSv, *p*-value < 0.05) but higher radiation exposure at the female gonads (6196.5 versus 1368.6 μSv, *p*-value < 0.05) (Table 1).

### Radiation exposure for fluoroscopic images

**Fig. 3:**
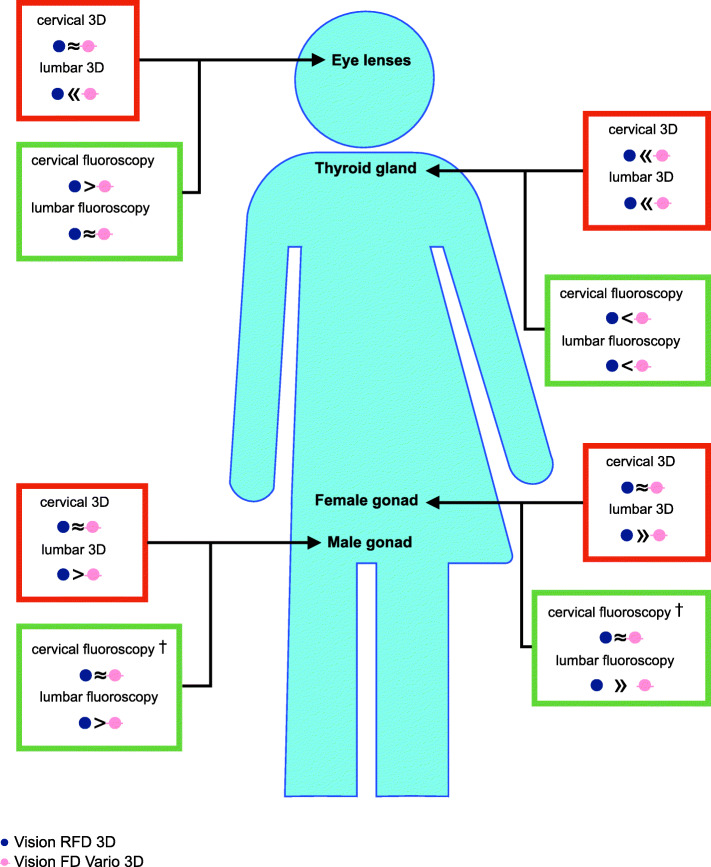
Comparative illustration of the radiation exposure of one cervical and one lumbar 3D image set and of one cervical and one lumbar standard fluoroscopic image using the Vision RFD 3D and Vision FD Vario 3D. » « Statistically significant difference. > < Numerical difference by more than 15% from each other without being statistically significant. ≈ Comparable radiation exposure of both C-arms. † Mean radiation exposure of both C-arms was below the lower detection limit

Table 2 shows the mean dosemeter readings for acquisition of cervical and lumbar standard fluoroscopic images without using beam collimation. In cervical fluoroscopic images, the dosemeters near/in the radiation field measured a numerically higher radiation exposure using the Vision RFD 3D at the eye lenses (10.6 versus 3.2 μSv) and a lower radiation exposure using the Vision RFD 3D at the thyroid gland (38.6 versus 50.6 μSv). In lumbar fluoroscopic images, the dosemeters at the female gonad showed a statistically significant higher radiation exposure using the Vision RFD 3D (132.9 versus 18.4 μSv, *p*-value < 0.05) (Fig. 3).

**Table 2:** Mean radiation exposures with standard deviation in μSv for acquisition of one cervical and one lumbar standard fluoroscopic image, respectively. The according exposure parameters are shown on the right. For each reading of a test series that was below the lower detection limit of the dosemeters, the value of the lower detection limit (44 μSv for film dosemeters) was used leading to the final estimation of a maximum radiation exposure of 0.11 μSv (44 μSv/400)

Type of dosemeter	Radiation exposure								X-ray tube voltage, current intensity	
	Film dosemeter						Electronic Personal Dosemeter			
**Site of dosemeter**	Eye lenses	Thyroid gland	Female gonad	Male gonad	C-arm generator	C-arm detector	Above lead apron ^a^	Under lead apron ^a^	Lateral projection	Anterior-posterior projection
**Cervical fluoroscopic image**										
Vision RFD 3D	10.6 ± 6.1	38.6 ± 11.4	< 0.11	< 0.11	0.39 ± 0.01^b^	0.57 ± 0.03	0.135	0.000	52 kV, 10.4 mA	58 kV, 12.0 mA
Vision FD Vario 3D ^c^	3.2 ± 0.2	50.6 ± 0.9	< 0.11	< 0.11	0.31 ± 0.01	0.58 ± 0.01	0.130	0.003	59 kV, 3.9 mA	65 kV, 4.8 mA
**Lumbar fluoroscopic image**										
Vision RFD 3D	0.15 ± 0.03	0.16 ± 0.02	132.9 ± 4.5^b^	0.72 ± 0.34	1.95 ± 0.03^b^	2.01 ± 0.02^b^	1.860	0.003	79 kV, 16.3 mA	74 kV, 15.7 mA
Vision FD Vario 3D ^c^	0.17 ± 0.01	0.26 ± 0.02	18.4 ± 9.3	0.27 ± 0.12	1.76 ± 0.03	1.76 ± 0.05	1.110	0.023	81 kV, 9.9 mA	74 kV, 8.5 mA

^a^Radiation exposures above and under a lead apron in μSv for acquisition of cervical and lumbar standard fluoroscopic images at the surgeon’s position directly next to the C-arm generator

^b^indicate *p*-values < 0.05 in comparison to the Vision FD Vario 3D

^c^Results of Klingler et al. [3]

The Vision RFD 3D showed at the site of the C-arm generator statistically significant higher radiation exposure in cervical and lumbar fluoroscopic images. Likewise, a higher radiation exposure using the Vision RFD 3D was measured at the site of the C-arm detector in lumbar fluoroscopic images (Table 2).

The calculated mean fluoroscopy time per image was 0.93 s (cervical spine as well as lumbar spine) for the Vision RFD 3D; for the Vision FD Vario 3D, a mean fluoroscopy time of 0.60 s (cervical spine) and 0.61 s (lumbar spine) was calculated.

The lead apron led to a protective reduction of at least 97% of the incident radiation. Current intensities were higher using the Vision RFD 3D on automated exposure (Table 2).

### Radiation exposure ratio between 3D image sets and fluoroscopic images

After determining radiation exposure of 3D image sets and standard fluoroscopic images, the number of standard fluoroscopic images was calculated whose radiation exposure corresponds to the radiation exposure of a 3D scan. The results showed that for example 56 standard fluoroscopic images on the cervical spine (Vision RFD 3D) or up to 93 standard fluoroscopic images on the lumbar spine (Vision RFD 3D) equal one 3D scan, depending on the dosemeter site (Table 3). This means that exceeding the number of fluoroscopic images specified in Table 3 results in a higher radiation exposure compared to a 3D scan with the respective 3D C-arm.

**Table 3:** Shown are the numbers of standard fluoroscopic images that equal a corresponding 3D scan in regard to the radiation exposure (calculated from the data of Tables 1 and 2)

	Eye lenses	Thyroid gland	Female gonad	Male gonad
**Cervical spine**				
Vision RFD 3D	27	56	n/a ^a^	n/a ^a^
Vision FD Vario 3D ^b^	91	87	n/a ^a^	n/a ^a^
**Lumbar spine**				
Vision RFD 3D	76	63	47	93
Vision FD Vario 3D ^b^	132	127	74	119

^a^Calculation was not feasible/meaningful because the mean radiation exposure was below/near the lower detection limit

^b^Results of Klingler et al. [3]

## Discussion

Advanced intraoperative 3D C-arms generate images in computed tomography like quality at lower radiation dose. However, existing implants such as screws cause reduced image quality due to artefacts. 3D C-arms with smaller dimensions facilitate improved maneuverability and usability in the operating room.

We measured the radiation exposure of the Vision RFD 3D using an Alderson phantom and compared it to previously reported results [3] of the identically investigated 3D C-arm device Vision FD Vario 3D.

### Radiation exposure for 3D image sets

The radiation exposure is, as to be expected, highest for lumbar 3D scans at the female gonads and for cervical 3D scans at the thyroid gland with both devices, because these organs are located in the direct course of the X-ray beam.

The Vision RFD 3D caused lower radiation exposures at all dosemeter sites in cervical 3D scans, as well as at the sites of eye lenses and thyroid gland in lumbar 3D scans. Thereby, the Vision RFD 3D offers almost twice the scan volume compared to the Vision FD Vario 3D (4096 versus 2097 cm^3^). At male and especially female gonads in lumbar 3D scans, however, the Vision RFD 3D showed substantially higher radiation exposures compared with the Vision FD Vario 3D (Fig. 3).

During one 3D scan, the Vision RFD 3D is able to display the whole cervical spine plus up to 2 thoracic spinal segments (about 8 segments in total). At the lumbar level, the whole lumbar spine plus 1 to 2 segments of the sacrum or up to 2 segments of the lower thoracic spine can be displayed (about 5 to 6 segments in total), depending on the patient’s individual anatomy. Using the Vision FD Vario 3D with the smaller flat-panel, about 6 segments of the cervical spine and approximately 4 segments of the lumbar spine can be displayed during a single 3D scan. The fact that a single 3D scan with the Vision RFD 3D can display a larger area is on the one hand helpful e.g. for longer instrumentations, but on the other hand, if only a short instrumentation is planned, the radiation exposure is unnecessarily increased.

### Radiation exposure for fluoroscopic images

Overall the dosemeters near/in the radiation field measured a higher radiation exposure using the Vision RFD 3D. This can be explained by the larger size of the flat-panel of the Vision RFD 3D compared to the Vision FD Vario 3D (2D fluoroscopic images: 900 cm^2^ versus 400 cm^2^). Thus, the Vision RFD 3D with the larger flat-panel is able to provide more information in one fluoroscopic image. If there is only a small region of interest (e. g. kyphoplasty of one vertebra), appropriate beam collimation should be used to adjust the field of view and to reduce radiation exposure. All fluoroscopic images in this study were performed without collimation**.**

The Vision RFD 3D showed statistically significant higher radiation exposures in cervical and lumbar fluoroscopic images at the site of the C-arm generator (that can be taken as approximation for the position of the surgeon). Likewise, a higher radiation exposure using the Vision RFD 3D was measured at the site of the C-arm detector in lumbar fluoroscopic images (Fig. 3). However, the absolute differences may not be regarded as very high.

The lead apron led to an efficient protective reduction of radiation (Table 2). Moreover, we recommend the use of further radiation protection equipment such as thyroid protection, lead glass goggles or mobile lead glass walls. Further radiation protection principles should be followed such as beam collimation and distance to the radiation source. Skin doses can be reduced by intermittent exposure, pulsed fluoroscopy and other dose reduction techniques [9].

### Comparison of radiation exposure between 3D image sets and fluoroscopic images

If exclusively the radiation exposure of the patient is considered, exceeding a certain number of fluoroscopic images can lead to a higher radiation exposure compared to one 3D scan (Table 3). However, considering the routine use of appropriate beam collimation in the area of interest, higher numbers of fluoroscopic images may certainly be reached.

Considering the measured radiation exposures, the numbers of standard fluoroscopic images that equal a corresponding 3D scan are less when using the Vision RFD 3D compared to using the Vision FD Vario 3D.

It is important to note that a 3D scan of the Vision RFD 3D allows a large scan volume over approximately 5 to 6 segments during lumbar scanning and 8–9 segments during cervical scanning. Unfortunately, the scan volume cannot be scaled down to reduce radiation exposure for cases, in which the larger field of view is not needed. It would therefore be desirable for the next generation of 3D C-arm devices with a large flat-panel to facilitate downsizing of the 3D scan volume. This feature would lead to a further reduction of radiation exposure to the patient e.g. in case of mono- or bilevel instrumentation procedures.

### Limitations

The use of a phantom has advantages such as the ability to reproduce the setting and the possibility of using ionizing radiation repeatedly. However, it can cause difficulties in transferring the results of studies with phantoms in real surgical situations with different patients and operations. Furthermore, we did not use thermoluminescence dosemeters inside the phantom that would have indicated the organ dose more accurately. It should also be mentioned, that the C-arm devices were set on automatic exposure control resulting in differing exposure parameters like tube voltages and current intensities.

The personal dosemeters are designed to estimate the effective dose by measuring a personal dose equivalent *H*_*p*_(d) in a determined tissue depth d. Therefore, the dosemeters are specified in their calibration, wearing position, angle of incidence etc. The dose equivalent *H*_*p*_(d) is composed by the exposure of the direct beam and the backscattered radiation of the body. To consider the backscattered radiation the personal dosemeters are calibrated with tissue equivalent phantoms. Varying materials and body sizes behind the dosemeters can lead to wrong estimations of the dose. Further, a higher angle of incidence than the required ±60° [IEC 62387] will cause an overestimation of the dose, while angles above 180° will lead to an underestimation of the dose.

Even if the measurement setup was not fully in line with the recommended use of the dosemeters at all times, the results are useful for comparative analysis and dose estimation. Considering that both investigations used the same setup, a relative comparison of both methods provides meaningful results.

Since the exact material compound of the C-arms is not known, it is difficult to state the difference in the backscattered radiation spectra compared to the expected spectra from human tissue. Hence, the absolute dose-values only give an estimate of the actual effective dose.

The doses measured above lead aprons are underestimated because lead scatters less radiation back than body tissue. Backscattering is part of the calibration for all personal doses. Due to the “simulated persons”, the backscattering of the dosemeters under the lead shield is also not completely correct, but this is probably less underestimated.

It has also to be considered that all fluoroscopic images in this study were performed without collimation, using two C-arms with different detector sizes and thus fields of view.

Image quality is a decisive criterion of a C-arm, but this was not the subject of this study. However, it can be deduced from clinical practice (without having conducted a systematic investigation) that the Vision RFD 3D provides improved image quality.

## Conclusion

Mobile 3D C-arms enable minimally invasive and precise pedicle screw placement by providing 3D image sets for intraoperative 3D imaging and navigation. However, any additional radiation exposure must be carefully considered, as there is basically no threshold dose below which ionizing radiation is negligible. Ionizing radiation should therefore be used as sparingly as possible. This study provides spinal surgeons with crucial information about the different radiation exposure of the respective spinal region caused by the mobile 3D-C-arm. In addition, the principles of radiation protection should be followed in order to further minimize radiation exposure.

## Data Availability

The dataset of the current study is available from the corresponding author on reasonable request.

## Abbreviations

2D: Two-dimensional
3D: Three-dimensional
